# Development of a Digital Patient Assistant for the Management of Cyclic Vomiting Syndrome: Patient-Centric Design Study

**DOI:** 10.2196/52251

**Published:** 2024-06-06

**Authors:** Gaurav Narang, Yaozhu J Chen, Nicole Wedel, Melody Wu, Michelle Luo, Ashish Atreja

**Affiliations:** 1 Rx.Health New York, NY United States; 2 Takeda Development Center Americas, Inc Lexington, MA United States; 3 UC Davis Health University of California, Davis Davis, CA United States

**Keywords:** cyclic vomiting syndrome, vomiting, vomit, emetic, emesis, gut, GI, gastrointestinal, internal medicine, prototype, prototypes, iterative, self-management, disease management, gut-brain interaction, gut-brain, artificial intelligence, digital patient assistant, assistant, assistants, design thinking, design, patient-centric, patient centred, patient centered, patient-centric approach, System Usability Scale, symptom tracking, digital health solution, user experience, usability, symptom, symptoms, tracking, monitoring, participatory, co-design digital health technology, patient assistance, patient experience, mobile phone

## Abstract

**Background:**

Cyclic vomiting syndrome (CVS) is an enigmatic and debilitating disorder of gut-brain interaction that is characterized by recurrent episodes of severe vomiting and nausea. It significantly impairs patients’ quality of life and can lead to frequent medical visits and substantial health care costs. The diagnosis for CVS is often protracted and complex, primarily due to its exclusionary diagnosis nature and the lack of specific biomarkers. This typically leads to a considerable delay in accurate diagnosis, contributing to increased patient morbidity. Additionally, the absence of approved therapies for CVS worsens patient hardship and reflects the urgent need for innovative, patient-centric solutions to improve CVS management.

**Objective:**

We aim to develop a digital patient assistant (DPA) for patients with CVS to address their unique needs, and iteratively enhance the technical features and user experience on the initial DPA versions.

**Methods:**

The development of the DPA for CVS used a design thinking approach, prioritizing user needs. A literature review and Patient Advisory Board shaped the initial prototype, focusing on diagnostic support and symptom tracking. Iterative development, informed by the design thinking approach and feedback from patients with CVS and caregivers through interviews and smartphone testing, led to significant enhancements in user interaction and artificial intelligence integration. The final DPA’s effectiveness was validated using the System Usability Scale and feedback questions, ensuring it met the specific needs of the CVS community.

**Results:**

The DPA developed for CVS integrates an introductory bot, daily and weekly check-in bots, and a knowledge hub, all accessible via a patient dashboard. This multicomponent solution effectively addresses key unmet needs in CVS management: efficient symptom and impacts tracking, access to comprehensive disease information, and a digital health platform for disease management. Significant improvements, based on user feedback, include the implementation of artificial intelligence features like intent recognition and data syncing, enhancing the bot interaction and reducing the burden on patients. The inclusion of the knowledge hub provides educational resources, contributing to better disease understanding and management. The DPA achieved a System Usability Scale score of 80 out of 100, indicating high ease of use and relevance. Patient feedback highlighted the DPA’s potential in disease management and suggested further applications, such as integration into health care provider recommendations for patients with suspected or confirmed CVS. This positive response underscores the DPA’s role in enhancing patient engagement and disease management through a patient-centered digital solution.

**Conclusions:**

The development of this DPA for patients with CVS, via an iterative design thinking approach, offers a patient-centric solution for disease management. The DPA development framework may also serve to guide future patient digital support and research scenarios.

## Introduction

Cyclic vomiting syndrome (CVS) is a disorder of gut-brain interaction with idiopathic etiology and is characterized by recurrent, stereotypical episodes of incapacitating nausea and vomiting [1]. Symptoms last from hours to days and episodes are interspersed with varying lengths of intervals with no or minimal symptoms [2-5]. CVS affects an estimated 2% of adults and 1.1% of children in the United States [2,6].

CVS remains a challenge to diagnose, treat, and manage given the nature of the condition (eg, exclusion diagnosis process without validated biomarker and heterogeneous presentation of symptoms among patients), a lack of awareness in the medical community, and patients’ lack of disease management knowledge [7-9]. Currently, there is no approved therapy for CVS to prevent or abort the vomiting attacks effectively. These factors lead to increased morbidity, decreased health-related quality of life (HRQOL), frequent leaves from work or school, increased health care resource use (eg, emergency department visits), and higher associated health care costs among patients living with CVS [10-13]. There also exist widespread communication challenges (eg, stigma) for patients with CVS, whose condition might be dismissed [14]. Patients have limited available resources to help them monitor symptomology, evaluate disease impact, track episode triggers, implement lifestyle modification, and combat stigma via better communication with health care providers (eg, emergency department doctors) and caregivers. These barriers suggest a high unmet need for a patient-centric and evidence-based tool to assist patients with CVS in disease management and communication, as well as to improve their quality of life.

Design thinking, which has been found to be an effective approach for achieving the desired acceptability and effectiveness of solutions by stakeholders involved in patient care, can address the complex needs of patients with CVS while following an iterative innovation-led process for better success [15].

Meanwhile, digital health technologies have emerged as effective solutions to help patients track their disease progression over time, including for patients with rare or orphan diseases [16-18]. Successful implementation of user-centered digital health interventions has improved patient’s quality of life across many common chronic conditions [19,20]. Some digital health apps and wearables for functional gastroenterological disorders such as Bowelle (Bowelle AB) and AbStats (Alpha Logic, Inc) have been developed; however, they only offer stand-alone tracking and symptomatic management, thus leaving a need for a multicomponent customized approach for patients with CVS [21].

This study aimed to develop for patients with CVS an artificial intelligence (AI)–powered digital patient assistant (DPA) that provides more than symptom tracking, using the iterative design principles of user design and user interface alongside the design thinking approach, which has been adopted successfully for the development of health care applications [15,22-24].

## Methods

### Overview

The design thinking approach, a user-centered problem-solving method, was adopted by this study’s team (ie, outcome researchers, technologists, and health care providers) to consolidate the perspectives and needs of end users (ie, patients) into technical features for a solution to manage the unique challenges for patients with CVS [22]. To implement the approach, several gaps need to be filled, notably: how it can be applied in health care contexts, how best to involve patients and other stakeholders in the design process, and the impact of design thinking on patient outcomes and experiences [15]. From late 2020 to early 2022, this study applied design thinking to develop the foundation for a DPA for patients with CVS.

**Figure 1:**
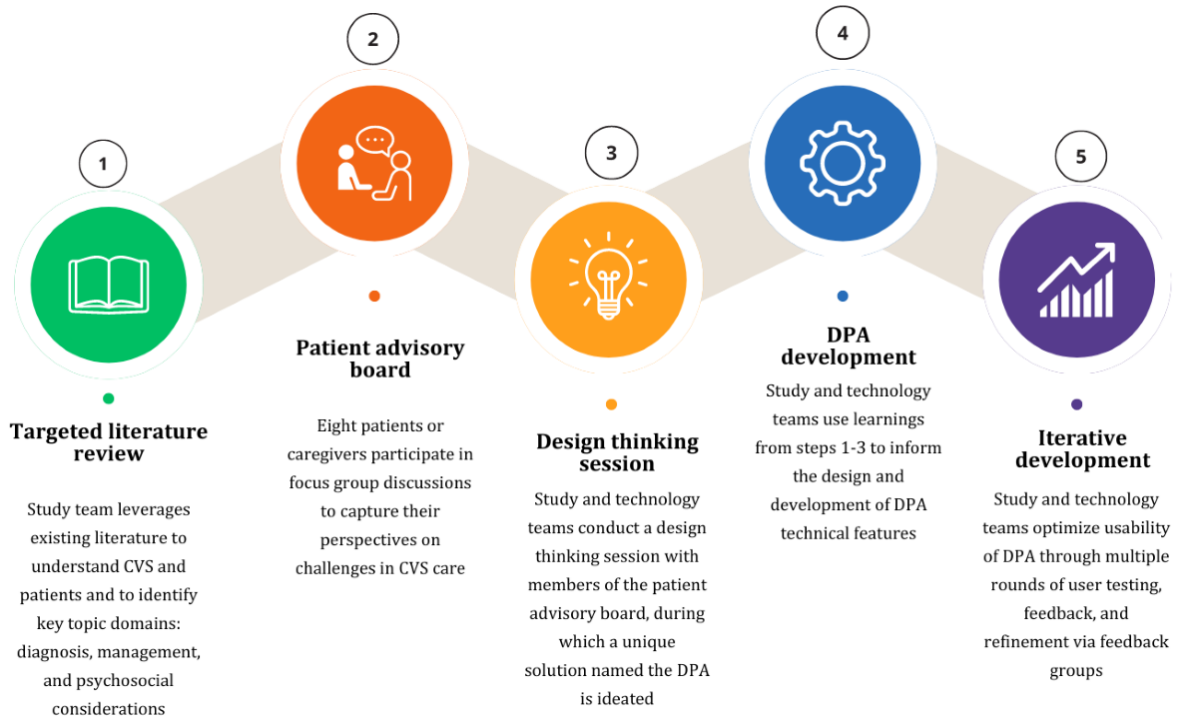
The 5-step approach to developing a DPA for CVS, starting with a TLR (1), followed by insights from a PAB (2), ideation in a design thinking session (3), technical development of the DPA (4), and iterative enhancement based on user feedback (5). CVS: cyclic vomiting syndrome; DPA: digital patient assistant; PAB: Patient Advisory Board; TLR: targeted literature review.

The execution of the design thinking framework includes 5 steps: defining, empathizing, ideating, prototyping, and testing; and they have been operationalized via 5 steps in this study (Figure 1). The first step (“defining”) was a targeted literature review (TLR) of important CVS topics conducted to compile a list of relevant domains to patients with CVS. It was followed by a second step (“empathizing”) wherein a Patient Advisory Board (PAB) identified common patient challenges and needs. In step 3 (“ideating”), a design thinking session was conducted to formulate a patient-centered design, and the DPA prototype was developed in step 4 (“prototyping”). Finally, iterative development (“testing”) was operationalized in step 5 with continued user feedback, rigorous feature evaluation, and enhancement.

### Ethical Considerations

This study’s design and methodology, involving patients with CVS and caregivers, were reviewed and approved by an institutional review board, ensuring compliance with human subject research standards. Informed consent was obtained from all participants, covering their primary participation and the secondary use of data. Privacy and confidentiality of participants were paramount, with measures implemented to anonymize and securely store all participant data. Participants were fairly compensated for their contributions, reflecting the ethical standards of this study. Additionally, this study was careful to ensure no identifiable images or data of participants were included in any publication or supplementary materials. In cases where such images or data were essential, explicit consent was obtained, and the relevant consent forms are available upon request.

### Step 1: TLR to Identify Key Topic Domains

This study’s team conducted a TLR on adult and pediatric patients with CVS, which was then used to develop a list of domains relevant to patients with CVS. Specifically, the TLR included articles related to CVS in epidemiology, pathology, economic and humanistic burden, as well as digital health intervention and design thinking, via searches at the National Center for Biotechnology Information literature databases and the sites of the Institute of Electronics and Electrical Engineers and the Centers for Disease Control and Prevention. A total of 26 studies were included and 3 domains were identified: diagnostic support, disease management, and psychosocial considerations.

### Step 2: PAB

As patient-centricity is at the core of the design thinking approach to address the unmet needs of patients with CVS, a PAB was conducted in 2020, not only to gain a comprehensive understanding of patient experiences and unmet needs but also to focus specifically on the 3 identified domains from the TLR.

The PAB included 5 individuals diagnosed with CVS, 2 caregivers, and 1 person who was both a patient with CVS and a caregiver to a child with CVS. CVS was diagnosed and documented by a physician. During the PAB, participants provided insights and shared details about their CVS experience and journey to diagnosis, symptoms, triggers, disease-related communication with health care providers, sources for educational materials used, disease management strategies, and the overall impact of CVS on their lives (Multimedia Appendix 1). They also shared perspectives on their relationships with their care team, understanding of the disorder, treatment guidance, and costs.

Direct patient insights gained from the PAB were critical in shaping the empathizing step of design thinking. Linking patient and caregiver insights to the 3 topic domains of diagnostic support, disease management, and social considerations would lead to the development of a solution that could improve the overall quality of life for individuals living with CVS.

### Step 3: Design Thinking Session

A design thinking session with this study’s team, the technology team (consisting of technical experts, including a product designer, a data scientist, and design technologists), and the PAB was implemented.

During the session, participants were first provided with an overview of the design thinking framework. To facilitate understanding of and empathy with the unique experiences and situations of patients with CVS, a diverse set of “patient personas,” that is, a fictional representation of a typical patient created to better understand the needs of the target patient population, was created based on the findings from the TLR and PAB. These personas were presented to patients and caregivers, who then provided input on the challenges that they faced. Health care providers shared insights into CVS symptomology and the considerations to present patient data to care teams, and technologists provided information on digital capabilities to address the needs of patients and providers and to shape the prospective digital solution. During the session, participants discussed their ideas, voted on the most promising solutions, and collaborated to refine the design of the selected digital health solution.

### Step 4: Development of the DPA

This study team and technology team developed the mockups and prototype of the DPA that included bots, a knowledge hub, and a patient dashboard by using iterative design principles of user design and user interface [25]. To address the need to understand patient-level disease status and experience over time, a chatbot framework was chosen. The initial version consisted of 2 chatbots: an “introductory bot” to obtain patient background and disease history, and a “check-in bot” to perform daily rapid tracking. The “introductory bot” enabled the creation of a patient profile, recording patient demographics and other characteristics, diagnosis, history of symptoms, triggers, CVS impact on life, and previous treatment. The “check-in bot” enabled regular check-ins on CVS-related symptoms, episodes, triggers, and life impact while also trying to minimize patient burden on data entry.

In the future, the DPA prototype will be integrated with other features of the Rx.Health platform to allow for disease monitoring by providers via an actionable dashboard in the electronic health record (EHR) system.

### Step 5: Iterative Development

The development of the DPA involved an iterative process of user feedback and improvements. A diverse group of participants, including adult patients with CVS, caregivers of pediatric patients with CVS, and health care providers, were recruited to test the initial version of the DPA. The testing involved semistructured interviews and real-time DPA testing using participants’ smartphones. Participants provided feedback on various aspects of the DPA, including information flow, wording, and appearance (eg, specific options in multi-choice questions).

Based on the feedback from the first round of testing, improvements were made to the DPA to address the issues identified. The updated version was then tested by another group of participants, including adult patients diagnosed with CVS and caregivers of pediatric patients with CVS. The testing involved the use of both the “introductory bot” and daily and weekly “check-in bots,” and participants provided real-time feedback on aspects of the DPA. To assess the usability and learnability of the DPA, these participants completed the System Usability Scale, a 10-item questionnaire with a 5-point Likert scale [26]. They also rated the bots’ question relevance and ease of use on a 5-point Likert scale.

## Results

### Overview

The DPA was developed through a multistep, iterative process, which started with the review of relevant literature that identified 3 critical domains (Textbox 1). Then, three key themes emerged from the PAB, including that (1) the disease journey from symptom onset to diagnosis is often lengthy and challenging in CVS, (2) the disease experience is heterogeneous among patients and can have a significant negative impact on patients’ outcomes, and (3) the disease management resources are scarce with a need for a multicomponent approach in addressing patient needs.

**Domains identified from targeted literature review (TLR).**

**Diagnostic support**

- Diagnosis criteria
- Absence of other organic causes
- Age of onset
- Frequency and severity of symptoms (eg, emesis)
- Concomitant psychological disorders (eg, anxiety or depression)

**Disease management**

- Understanding patients’ patterns of symptoms or episodes
- Understanding the use of medical services or medications
- Satisfaction with care

**Psychosocial considerations**

- Impact on patients
  Adult and pediatric: low health-related quality of life (HRQOL) and productivity loss at work
  Pediatric: delayed childhood development, school absences, and poor academic performance
- Impact on caregivers: productivity loss at work
- Lack of social support

### Design Thinking Session

**Figure 2:**
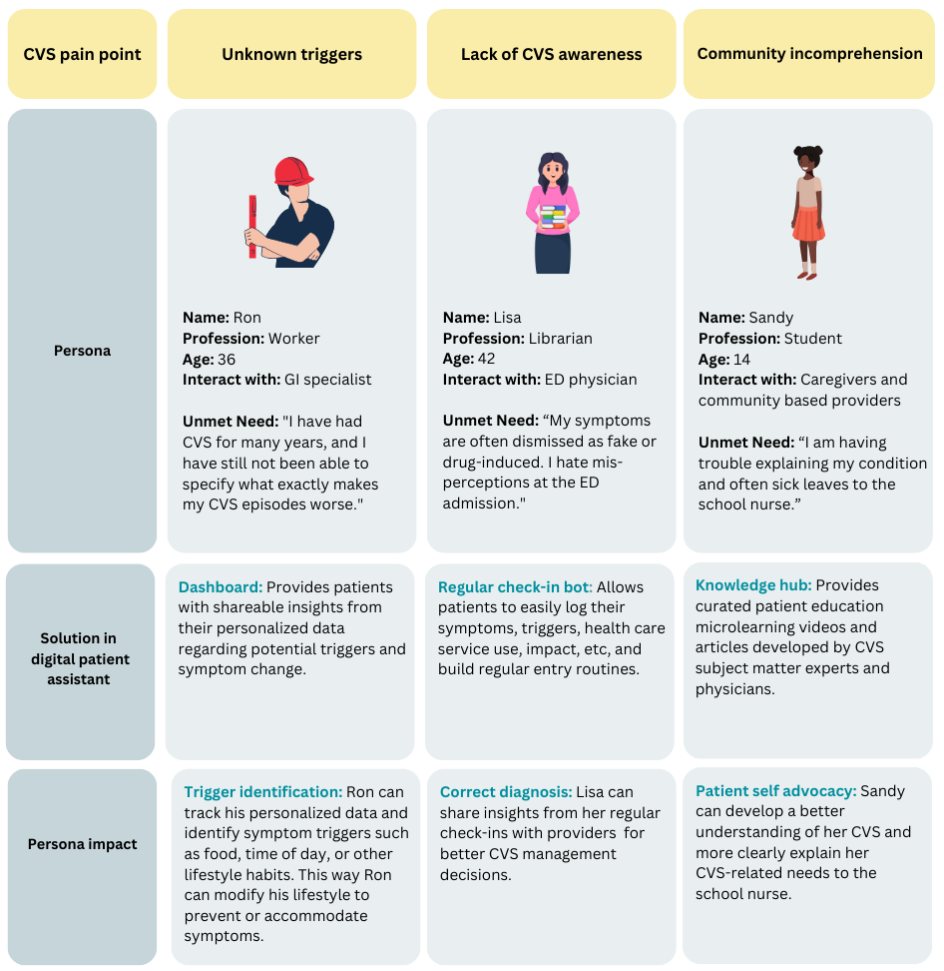
Illustration of pain points in CVS management linked to targeted solutions offered by the DPA, demonstrated through personas of varying demographics and needs, and showcasing the personalized impact of the DPA features on each individual’s journey. CVS: cyclic vomiting syndrome; DPA: digital patient assistant; ED: emergency department; GI: gastrointestinal.

During the design thinking session, participating PAB members discussed the different challenges faced by the personas with CVS (Figure 2), and the technology team shared 3 potential digital solutions: CVS Share (a social networking site linking patients to support resources), Hospital@home (remote patient management through telehealth check-ins and devices), and the DPA. Following a vote from this study’s team and PAB members, the DPA was chosen for development because of its versatility, low cost to build and implement, and ability to address all pain points brought up during the session with features such as the inclusion of electronic patient-reported outcome questionnaires that assess symptomology, quality of life, and mental health well-being. The technology team presented a mock patient dashboard with data from the questionnaires, which could provide valuable insights into symptom tracking and episode trigger identification. Along with the provision of educational resources on CVS via a knowledge hub, the dashboard addresses long-standing challenges in CVS management. The digital solution prototype was built on the Rx.Health digital automation platform, which will eventually integrate the patient-friendly DPA with disease monitoring features for providers.

### Development of the DPA

The results from earlier development steps were integrated into the first iteration of the DPA. This prototype was comprised of an introductory bot, a check-in bot, a knowledge hub providing access to CVS-related articles and microlearning videos, and a patient dashboard showing a snapshot of potential triggers, symptoms, episode frequency, DPA use, and correlation with vital health metrics. The DPA was designed to comprehensively address the identified needs of patients, caregivers, and providers, and help patients with better disease and care management.

### Iterative Development

At a user feedback session following the initial version of the DPA, patients and caregivers found the check-in bot to be both relevant and user-friendly. The participants rated the bot’s question relevance at 4.17 and ease of use at 4.3 on a 5-point Likert scale ranging from 1 (strongly disagree) to 5 (strongly agree). Participants also confirmed the need for a multicomponent solution to better support patients with CVS with disease monitoring and communication.

Meanwhile, the participants also suggested further improvements in several areas. First, concerning the check-in bot, participants were less willing to use it regularly (rated 2.75) but nonetheless expressed a willingness to do so if it was prescribed by health care providers. They also requested that the frequency of check-ins be modified to allow for self-scheduled or symptom-based check-ins. Third, participants suggested including additional questions about symptoms and triggers and explanations for certain terms. Finally, providers suggested adding information on complementary treatment options and care management locations to the knowledge hub, using empathetic language, and continuing the user feedback integration throughout the development process.

**Figure 3:**
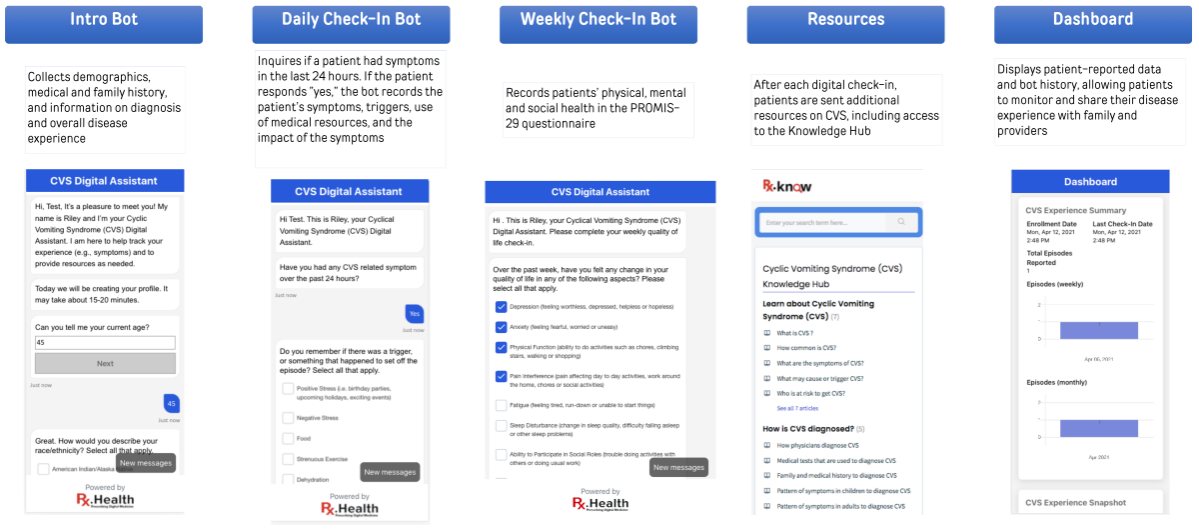
Components of the DPA for CVS, showcasing the intro bot for initial data gathering, daily and weekly check-in bots for ongoing symptom and impacts monitoring, a resources section for patient education, and the dashboard for data visualization and tracking. CVS: cyclic vomiting syndrome; DPA: digital patient assistant.

The DPA was further modified in response to the feedback and to ensure alignment with identified CVS-related domains and themes. Significant changes included modifications to the bots’ frequency of check-ins by creating both daily and weekly check-in bots (Figure 3). The latter would ask HRQOL questions. Additionally, AI features such as intent recognition and data syncing were incorporated into the DPA to relieve patient burden.

The second phase of user feedback yielded positive results, with an average System Usability Scale score of 80 out of 100, which indicated a high level of ease of use. Participants provided real-time feedback on various aspects of the DPA, including the length of each section, topic comprehensiveness, and the appropriateness of questions. They also suggested several other areas for improvement (eg, more free text options, additional answer choices, and clarification of health literacy terms).

Updates following the second phase of feedback included modifying the daily check-in bot to start with a yes or no question on whether the user experienced any CVS symptom in the past 24 hours so that those without symptoms would not be asked further questions for that day. The technical features of the final version of the DPA are presented in Table 1.

**Table 1:** DPA^a^ technical features by domains, themes, and unmet patient and caregiver needs.

Domain and theme		Unmet patient or caregiver needs	Technical features of the DPA
**Diagnostic support and psycho social considerations**			
	Disease experience is largely variable and often negatively affects outcomes	An efficient way to track symptoms, triggers, and impacts	Weekly and daily check-in bots: The bots allow patients to record symptoms and understand potential triggers including diet, anxiety sources, physical activity, etc, via the patient dashboard. Auto-termination: Only patients who report having symptoms in the past 24 hours complete all the questions from the daily bot. If the patient has no symptoms, the bot automatically terminates for that day. Intent recognition: Frequently entered free-text responses will appear as structured options. Data sync: The bot remembers and preselects previously selected responses and provides personalized questions.
	The CVS^b^ journey to diagnosis is often long and difficult	CVS diagnosis and management information	Knowledge hub: This provides patients and caregivers with access to CVS-related information regarding diagnosis and management via infographics, microlearning videos, articles, etc, that they can also share with others.
**Disease management**			
	There are a variety of disease management options but patients desire proven effectiveness	An evidence-based digital health solution to aid with disease management and tracking	Platform approach: The DPA serves the needs of patients with CVS by providing access to the symptom tracking information in a dashboard that can be shared with others via email, as well as a knowledge hub. Future anticipated studies may validate the DPA’s effectiveness and explore its integration into a digital health toolkit that also includes integration of dashboard data with EHR^c^ systems for provider review.

^a^DPA: digital patient assistant.

^b^CVS: cyclic vomiting syndrome.

^c^EHR: electronic health record.

## Discussion

### Principal Learnings

The DPA represents the development of an innovative solution in the realm of patient-centered health care technology tailored for individuals with CVS, who face significant unmet needs in their disease experience [5]. The iterative development process of the DPA used a design thinking approach that placed a strong emphasis on engagement with patients and caregivers throughout the entire process. For example, patient feedback regarding the data entry burden led to the implementation of AI and machine learning features, such as saving frequently selected responses, as well as switching from a daily to a weekly HRQOL questionnaire.

Patient preference for a convenient platform was reflected in the choice of a chatbot as the primary mode of communication for the DPA. Similarly, the patient dashboard was designed to be easily accessible to patients through smartphone technology, and a dashboard accessible to health care providers through EHR integration is planned as part of future development to support the monitoring of patient data. Integration into the EHR system may help to relieve the burden on health care providers by presenting all patient data in a holistic platform. The use of the DPA supports patients in being experts on their own health and encourages health care providers to work collaboratively with patients to develop management plans that align with their goals and preferences.

The DPA has the potential to overcome the limitations of stand-alone symptom-tracking apps for functional gastrointestinal disorders through additional features such as educational modules with behavioral suggestions through the knowledge hub. In other disease areas, these features have supported improved health outcomes, and could also inform patients with relevant knowledge to support themselves in various settings [27,28]. Furthermore, the multicomponent approach of the DPA offers a more comprehensive solution for addressing the varying needs of patients compared to stand-alone services. The DPA integrates not only symptom tracking and education, but also assists in treatment management through data sharing with the patient’s care team currently by allowing patients to share their dashboard data and, in future development, by allowing that data to be integrated into the EHR system.

The DPA received positive feedback from most patients and caregivers regarding its relevance and ease of use. However, it was observed that the uptake and use of the DPA may be influenced by expert endorsement. Patients are more likely to use it regularly if recommended by their health care providers. Further investigation is necessary to understand the rationale behind patient perceptions and to identify any potential barriers to use, such as concerns about data privacy or a lack of perceived need. Until then, health care providers and clinical staff should play a critical role in encouraging the use of the DPA as a tool for collecting information to improve disease management for patients with CVS and their caregivers.

### Limitations

Due to the limited number of patients who tested the DPA and the limited number of measures, it was challenging to determine any associations between the DPA features and patients’ engagement levels or quality of life. Future research is needed to assess the long-term impact of the DPA on health outcomes, quality of life, and satisfaction in patients with CVS. Further demographic and clinical data are necessary to understand the influence of socioeconomic, cultural, and disease severity factors on the usability, adoption, and effectiveness of the DPA. Future explorations may involve DPA testing in more patients (eg, to evaluate the usability, relevance, patient engagement rate, and outcomes) via a partnership with patient communities. Such exercises may help assess the DPA in user scenarios beyond symptom and trigger tracking.

### Conclusions

This paper presents a multistep framework for developing a digital assistant for patients with CVS. Patients are inclined to embrace the DPA as it is both relevant and user-friendly, especially when it is prescribed by their physicians. This approach demonstrates the potential to enhance patient engagement and readiness while effectively addressing disease-specific needs. Future development of the DPA (eg, integration to EHR) may consider the inclusion of the ability to prescribe the DPA to patients’ mobile phones and provision for a wider range of users (eg, providers, health systems, and patient advocacy groups) to assess the impact on disease management and patient outcomes. Finally, the DPA development process outlined here offers the potential to serve as a template for designing, testing, deploying, monitoring, and scaling other innovative, patient-centered digital health applications.

## Appendix

Multimedia Appendix 1 Patient Advisory Board (PAB) worksheets.

## Abbreviations

AI: artificial intelligence
CVS: cyclic vomiting syndrome
DPA: digital patient assistant
EHR: electronic health record
HRQOL: health-related quality of life
PAB: Patient Advisory Board
TLR: targeted literature review

## Data Availability

The data sets, including the redacted study protocol, redacted statistical analysis plan, and individual participants’ data supporting the results reported in this paper, will be made available within 3 months from initial request to researchers who provide a methodologically sound proposal. The data will be provided after its deidentification, in compliance with applicable privacy laws, data protection, and requirements for consent and anonymization.
