# Supplementary material for: Development of a Digital Patient Assistant for the Management of Cyclic Vomiting Syndrome: Patient-Centric Design Study
Source: JMIR Form Res. 2024 Jun 6;8:e52251. doi: 10.2196/52251 (PMC11190623; doi:10.2196/52251)
Supplement: Multimedia Appendix 1 [file formative_v8i1e52251_app1.docx]

## Appendix A. Patient Advisory Board (PAB) worksheets.

PAB participants shared their experience as patients/caregivers through the Treatment Worksheet, Ranking Worksheet, Attribute Worksheet, Relationship Worksheet, and Treatment Communication Worksheet. Responses to the Treatment Worksheet provide the participants’ used treatments and their working knowledge of treatments for CVS symptoms including benefits, drawbacks, and whether a treatment is reactive or proactive. The Ranking Worksheet allows participants to share insights on factors that influence treatment choice including treatment costs, episode intensity, side effects, and episode frequency. The Attribute Worksheet asks participants to choose and rank desirable treatment attributes including convenience, fast-acting, etc. The Relationship Worksheet ascertains the type of relationship between the patient and their providers by comparing them to well-known relationship archetypes.

| 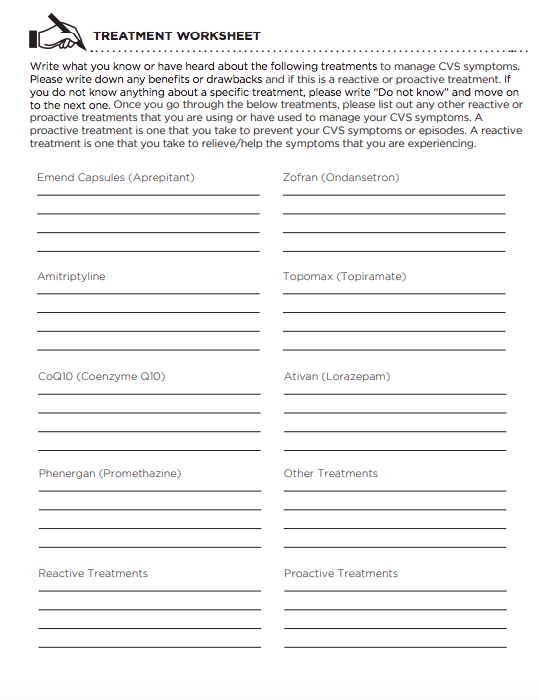 | 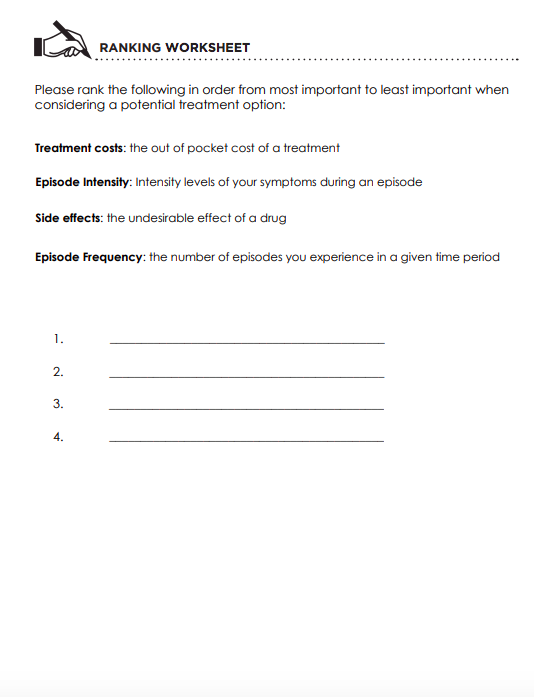 |
| --- | --- |
| 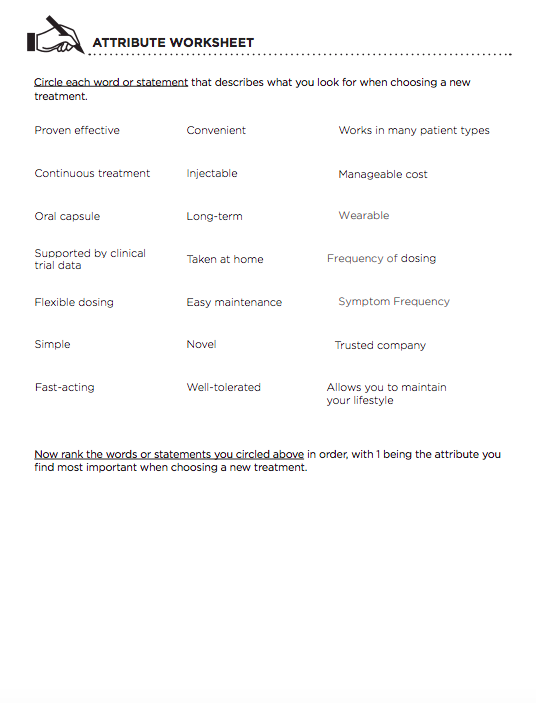 | 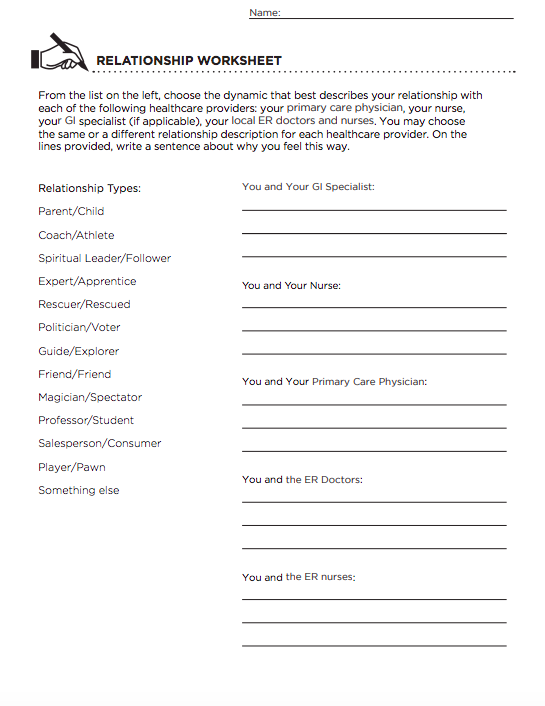 |
| 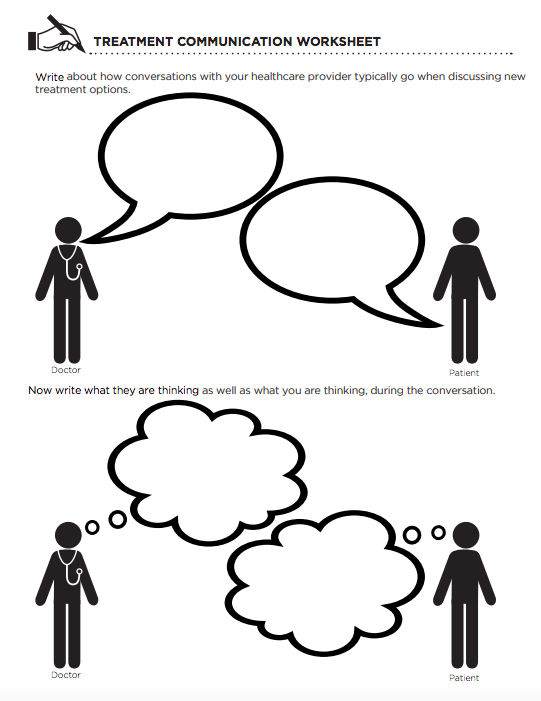 |  |
